# The Effects of Rape Residue Mulching on Net Global Warming Potential and Greenhouse Gas Intensity from No-Tillage Paddy Fields

**DOI:** 10.1155/2014/198231

**Published:** 2014-07-22

**Authors:** Zhi-Sheng Zhang, Cou-Gui Cao, Li-Jin Guo, Cheng-Fang Li

**Affiliations:** ^1^College of Plant Science and Technology, Huazhong Agricultural University, No. 1 Shizishan Street, Hongshan District, Wuhan, Hubei 430070, China; ^2^Key Laboratory of Crop Ecophysiology and Farming System in the Middle Reaches of the Yangtze River, No. 1 Shizishan Street, Hongshan District, Wuhan, Hubei 430070, China

## Abstract

A field experiment was conducted to provide a complete greenhouse gas (GHG) accounting for global warming potential (GWP), net GWP, and greenhouse gas intensity (GHGI) from no-tillage (NT) paddy fields with different amounts of oilseed rape residue mulch (0, 3000, 4000, and 6000 kg dry matter (DM) ha^−1^) during a rice-growing season after 3 years of oilseed rape-rice cultivation. Residue mulching treatments showed significantly more organic carbon (C) density for the 0–20 cm soil layer at harvesting than no residue treatment. During a rice-growing season, residue mulching treatments sequestered significantly more organic C from 687 kg C ha^−1^ season^−1^ to 1654 kg C ha^−1^ season^−1^ than no residue treatment. Residue mulching significantly increased emissions of CO_2_ and N_2_O but decreased CH_4_ emissions. Residue mulching treatments significantly increased GWP by 9–30% but significantly decreased net GWP by 33–71% and GHGI by 35–72% relative to no residue treatment. These results suggest that agricultural economic viability and GHG mitigation can be achieved simultaneously by residue mulching on NT paddy fields in central China.

## 1. Introduction

China is the largest rice-producing country in the world, with a gross sown area of 29.6 million ha in 2009 [1]. CH_4_ emissions from Chinese rice fields during the 2000 rice-growing season have been estimated to be 7.4 Tg, constituting approximately 29% of global CH_4_ emissions from rice cultivation [2]. Annual N_2_O emissions from rice fields in China have been estimated to be 91 Gg nitrogen (N), of which 50 Gg N is emitted during rice-growing seasons [3]. Furthermore, mean soil CO_2_ fluxes from paddy fields of subtropical China have been estimated to be 178.5–259.9 mg m^−2^ h^−1^ [4], which are far more than mean fluxes of N_2_O (6.0–74.3 *μ*g m^−2^ h^−1^) [5] and CH_4_ from paddy fields in China (0.5–32.3 mg m^−2^ h^−1^) [6]. In this way, mitigating GHG emissions from paddy fields in China is an important means of addressing the issue of climate change and developing sustainable agriculture.

China produces approximately 750 million tons of crop residues annually [1]. Farmers generally burn crop residues in their fields to reduce the time and expense of handling them, causing environmental pollution. For this reason, farmers are encouraged to return residues to their fields after harvesting, to stop burning them, and to improve the sustainability of agriculture. The soil C pool depends on the balance between C input and output. The use of crop residues can increase C input to soil and so improve soil C sequestration. However, this undoubtedly provides readily available C and N substrates, thus increasing GHG emissions [7]. Increases in GWP caused by increased GHG emissions from the use of crop residue application may significantly offset the mitigation benefits of soil C sequestration [8]. Effective measurement of crop residue returns and the resulting mitigation of climatic impacts requires a complete perspective of the impacts of returning crop residues on GHG emissions and soil C sequestration [9].

GWP is a simplified index based on radiative properties introduced to assess the potential impacts of GHG emissions on the climate system [10]. To estimate GWP, CO_2_ is typically regarded as the reference gas, and an increase or decrease in CH_4_ and N_2_O emissions is converted into CO_2_ equivalents through their GWP. A positive GWP represents a net source of CO_2_ equivalents, and a negative value indicates a net sink of atmospheric GHGs. Net GWP reflects a complete understanding of agriculture's impact on radiative forcing and is calculated by the balance between SOC storage and N_2_O and CH_4_ emissions [10, 11]. In addition, GHGI relating agricultural practices to GWP is calculated by dividing GWP by crop yield. A positive GHGI value indicates a net source of CO_2_ equivalents per kilogram of yield, whereas a negative value indicates a net sink of GHG in soil [9]. Although the effects of crop residue incorporation on GWP, net GWP, or GHGI from paddy fields or uplands have been reported [10, 11], little information is available on the effects of crop residue mulching on NT rice fields on GWP, net GWP, and GHGI.

NT rice cultivation has drawn increasing amounts of interest in China due to saving time and labor and preventing the soil erosion [12]. Central China is one of the country's major rice-producing regions, comprising 28% of the total area of cultivated rice in China [13]. Recently, NT practices have become increasingly popular in this region. This inevitably increases the amount of crop residue. So, the establishment of a government policy favors these crop residues returned to the field. However, crop residue mulching on the soil surface of NT rice fields may have different effects on GHG emissions than residue incorporation. Moreover, although a great amount of field measurements have focused on the effects of crop residue returning on GWP, or net GWPs or GHGI from paddy fields based on CO_2_, CH_4_, and N_2_O flux data [14–16], to our knowledge, a few simultaneously investigated the effects of crop residue returning on these three indices from paddy fields, especially from NT paddy fields. In this way, the present study aims to (1) quantify GHG emissions under different oilseed rape residue mulching regimens in central China during the 2010 rice-growing season and (2) assess GWP, net GWP, and GHGI.

## 2. Materials and Methods

### 2.1. Site Description

This field trial was established in an experimental farm (Zhonggui Village, 29°55′ N, 115°30′ E, Wuxue City, Hubei, China), which belongs to Extend Service Center of Agricultural Technology of Wuxue Agricultural Bureau, Hubei. This experimental area has a humid mid-subtropical monsoon climate with an average annual temperature of 16.8°C and a mean annual precipitation of 1360.6 mm. For the 5 years prior to study initiation, most of the rainfall occurred between April and August. The soil in the experimental site is clay loam and is classified as anthrosol [17]. The main soil properties (0–20 cm depth) in the site sampled in October 2006 are as follows: pH (extracted by H_2_O; soil : water = 1.0 : 2.5), 6.58; organic C, 17.9 g kg^−1^; total N, 2.21 g kg^−1^; NO_3_^−^–N, 3.78 mg kg^−1^; NH_4_^+^–N, 13.15 mg kg^−1^; total P, 0.53 g kg^−1^; and soil bulk density, 1.26 g cm^−3^.

### 2.2. Experimental Design

The experiment was designed as a randomized complete block with three replicates (45 m^2^) and was established in October 2006, three years before the study began. The variety of mid-season rice planted was Liangyoupeijiu (*Oryza sativa* L.). The experimental site was cultivated with a rape-rice rotation for 30 years prior to October 2006, where rice was transplanted with conventional tillage (soil is commonly tilled to a 10 cm depth by hand and then moldboard plowed to a depth of 20 cm by animal power) from May to October each year and rape was planted with conventional tillage from October to May the following year. The experiment included four treatments: (1) no oilseed rape residue mulching (0 RRM), (2) 3000 kg DM ha^−1^ oilseed rape residue mulching (3000 RRM), (3) 4000 kg DM ha^−1^ oilseed rape residue mulching (4000 RRM), and (4) 6000 kg DM kg ha^−1^ oilseed rape residue mulching (6000 RRM) on the surface of the soil in NT rice fields. For the treatment of 0 RRM, oilseed rape residues were removed and not returned to the field. In the residue mulching treatments, dried oilseed rape residues were chopped to approximately 5–7 cm in length according to local conventional practice. The C/N ratio of the oilseed rape residue was 48.5.

Preemergent herbicides (20% paraquat) were used to control weeds on June 3, 2010. The experimental samples were then submerged. No soil disturbances appeared in any of the plots immediately after oilseed rape was harvested. Rice seeds were sown manually at a rate of 22.5 kg ha^−1^ on June 6, 2010 and harvested on October 17, 2010. Dried oilseed rape residues were mulched immediately on the rice fields on June 11, 2010. Commercial inorganic N-phosphorus- (P-) potassium (K) fertilizer (15% N, 15% P_2_O_5_, and 15% K_2_O), urea (46% N), single superphosphate (16% P_2_O_5_), and potassium chloride (60% K_2_O) were used to supply 210 kg N ha^−1^, 135 kg P_2_O_5_ ha^−1^, and 240 kg K_2_O ha^−1^ during the rice-growing season. N fertilizers were supplied at a rate of 84 kg N ha^−1^ in the form of basal fertilizers immediately before crop residues were applied. The remaining N fertilizers were split into two doses of 63 kg N ha^−1^ on June 26 and July 21, 2010. P and K fertilizers were only used as basal fertilizers immediately after seeding. The plots were regularly irrigated based on local conventional irrigation-drainage practices during rice-growing seasons. Air temperature data in the experimental site were collected from the weather station in Wuxue city.

### 2.3. Soil Sampling and Analysis of SOC and Bulk Density

Paddy soil samples (0–20 cm depth) were collected to determine total organic C using a soil sampler with a diameter of 5 cm at five random positions in each plot 1 day before the preemergent herbicides were applied and immediately after rice was harvested. Total organic C in the 0–20 cm soil layer was determined by dichromate oxidation and titration with ferrous ammonium sulfate [18]. Soil bulk density for the 0–20 cm soil layer was determined by the method of Bao [18]. Soil samples were collected to measure bulk density using metallic cores (5.3 cm in diameter and 20 cm tall) with three replicates per plot. SOC density (kg C ha^−1^) for the 0–20 cm soil layer was computed from the SOC concentration and the soil bulk density described by Lu et al. [19]. SOC sequestered during a rice-growing season can be calculated as the differences of SOC density between the end and beginning of the experiment.

### 2.4. Measurement of Rice Grain Yields

The rice grains harvested in 2010 were measured at three random positions in each plot using a 1 m × 1 m frame. Rice grains were weighed and adjusted to have 14% moisture content.

### 2.5. Measurement of CO_2_, CH_4_, and N_2_O

In this study, soil CO_2_ fluxes from paddy fields were monitored with a Li-6400 portable photosynthesis analyzer (LI-COR Biosciences, U.S.). Soil fluxes were measured over the course of 2 h between 9:00 and 11:00 (a representative time of day in this region, according to Lou et al. [20]). Soil CO_2_ fluxes were measured at the same time as CH_4_ and N_2_O fluxes. Soil CO_2_ flux was determined every 20 s for 180 s. Three measurements were performed for each plot on each sampling day. The soil CO_2_ flux value considered in this study was the average of three individual measurements and is here expressed as mg m^−2^ h^−1^.

Fluxes measurements of CH_4_ and N_2_O from all plots were conducted simultaneously by closed steel cylinders with a diameter of 58 cm and a height of 110 cm [21]. Each steel cylinder covered four hills of rice. Two permanent base rings were placed below the water level to create a seal in each plot. The steel cylinders were placed temporarily on these rings to measure gas fluxes. The installed equipment on the chambers was detailed by Li et al. [21]. The gases in the chamber were then drawn with a syringe and immediately transferred into a 25 mL vacuum glass container. Four gas samples were taken using 25 mL plastic syringes at intervals of 0, 8, 16, and 24 min after closing the chambers. The CH_4_ and N_2_O fluxes were measured between 9:00 and 11:00 am. The gas samples were collected 1 day after oilseed rape residue mulching was conducted until rice harvesting. The gas samples were collected 20 times during the rice-growing seasons based on climate conditions and N fertilization.

The concentrations of CH_4_ and N_2_O were analyzed with a gas chromatograph meter equipped with an electron capture detector for N_2_O analysis and a flame ionization detector for CH_4_ analysis, following the method described by Li et al. [21]. The CH_4_ and N_2_O fluxes were calculated based on changes in their concentrations throughout the sampling period, being estimated as the slope of linear regression between concentration and time [21].

Seasonal total GHG emissions were calculated for each plot by linearly interpolating gas emissions during the sampling days based on the assumption that the measured fluxes represented the average daily fluxes [21].

### 2.6. GWP, Net GWP, and GHGI

In the present study, CO_2_ emission was based only on soil flux measured between rows of rice plants. Moreover, CO_2_ emissions and consumption resulting from plant respiration and photosynthesis were not considered during the calculation of GWP. Under conventional rice-growing conditions, CO_2_ emissions and consumption caused by plant respiration and photosynthesis are balanced [14]. In this way, CO_2_ emissions from rice respiration are not considered when computing GWP from agriculture. Consequently, the GWPs (kg CO_2_ equivalents ha^−1^) of different treatments were calculated using

(1) $$\begin{array}{c} \text{GWP}={\text{CO}}_{2}+{\text{CH}}_{4}\times 25+{\text{N}}_{2}\text{O}\times 298. \end{array}$$

Based on a 100-year time frame, the GWP coefficients of CH_4_ and N_2_O are 25 and 298, respectively, when the GWP value of CO_2_ is assumed to be 1 [22].

Soil CO_2_ is released by the decomposition of crop residues and soil organic C. It is affected by changes in agricultural management. Changes in soil organic C are the product of soil C sequestration and soil CO_2_ emissions. In this way, CO_2_ flux from soil is inherently accounted for in changes in soil organic C [23]. Robertson et al. [10] and Shang et al. [11] suggested that changes in soil organic C should be measured, thus accounting for the GWP of soil. Here, net GWP was calculated using [11]

(2) $$\begin{array}{c} \text{Net GWP}={\text{CH}}_{4}\times 25+{\text{N}}_{2}\text{O}\times 298-\Delta \text{TOCD}\times \frac{44}{12}. \end{array}$$

Thereafter, GHGI (kg CO_2_ equivalents kg^−1^ grain yield) was calculated by dividing net GWP by rice grain yield using [9, 11]

(3) $$\begin{array}{c} \text{GHGI}=\frac{\text{Net GWP}}{\text{grain yield}}. \end{array}$$

### 2.7. Data Analysis

SPSS 16.0 analytical software package (SPSS Inc., USA) was used for all statistical analyses. A one-way ANOVA of SPSS 16.0 was used to determine the effects of residue mulching on total organic C, organic C density, organic C sequestration, GHG emissions, GWP, and GHGI. Individual means were compared based on the least significant difference test. Only the means that were statistically different at *P* ≤ 0.05 were considered different.

## 3. Results and Discussion

### 3.1. Total Organic C and Organic C Sequestration

Returning crop residues to the field is highly recommended as a means of increasing soil organic C concentration and storage in rice-based cropping systems [24].  Table 1 shows that residue mulching had significant effects on total organic C and organic C sequestration from NT paddy fields. Total organic C concentration and density at harvesting exhibited a tendency to increase as the amount of mulch used increased. Residue mulching treatments significantly increased total organic C concentrations at harvesting by 1.50 g kg^−1^ to 2.96 g kg^−1^, equivalent to organic C density of 3780 g kg^−1^ to 7459 g kg^−1^. During a rice-growing season, the treatments of 3000 RRM, 4000 RRM, and 6000 RRM significantly sequestered more organic C than no residue treatment, by 687 kg C ha^−1^ season^−1^, 1257 kg C ha^−1^ season^−1^, and 1654 kg C ha^−1^ season^−1^, respectively. The positive effects of residue mulching on soil organic C concentration and sequestration reflect considerable C supplementation to soil under these regimens. Similarly, a study performed in India showed that, in a single rice-growing season, the use of rice straw in a site that had been cultivated for 4 years significantly increased soil organic C concentration and sequestration [15]. The soil organic C sequestration caused by residue mulching in an NT rice system is attributed to the fact that the soil was flooded for 4 months and to the high biomass production of rice (Table 3). When the concentration of O_2_ under submerged conditions is very low, even the moderate oxygen demands of microbial activity go unmet if large pores are filled with water, decreasing decomposition rates [15]. Sahrawat [25] found that flooded rice soil exhibited better accumulation of organic matter than aerobic soil because of incomplete decomposition of organic materials and decreased humification of organic matter under flooded conditions.

In the present study, soil organic C sequestration increased as the amount of mulching increased (Table 1), indicating that long-term cultivation of crop leads to deficits in soil organic C in central China. In this way, short-term residue application was found to promote accumulation of soil organic C.

### 3.2. Rice Grain Yield

In general, long-term application of organic residues and with chemical NPK fertilizers can increase crop yields of rice-based cropping systems because of improved soil fertility [26]. In the present study, residue mulching was found to have no significant effect on rice yields during a 4-year field experiment (Table 3). N fertilizers were applied at a rate of 210 kg N ha^−1^ during the rice-growing season. This is, on average, 28% higher than the recommended rates for Chinese cereal production (150−180 kg N ha^−1^, Zhu and Chen [27]). Therefore, although residue mulching improved soil fertility, high N input could satisfy the N demands of rice, weakening the effects of residue mulching on rice yields [28]. Similarly, during a 3-year field experiment in Jiangsu province, China, Ma et al. [29] found no significant difference in rice grain yields between plots treated with residue and untreated plots. However, Ge et al. [30] found maize straw to have positive effects on rice yields during a 3-year field experiment. Further study on the short-term effects of crop residue application on rice yields is necessary.

### 3.3. GHG Emissions

**Figure 1:**
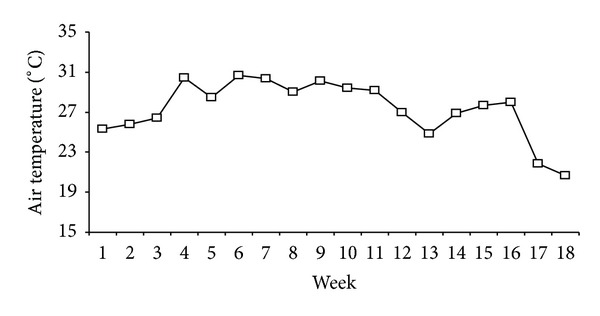
Changes in mean weekly air temperature during the rice-growing season.

**Figure 2:**
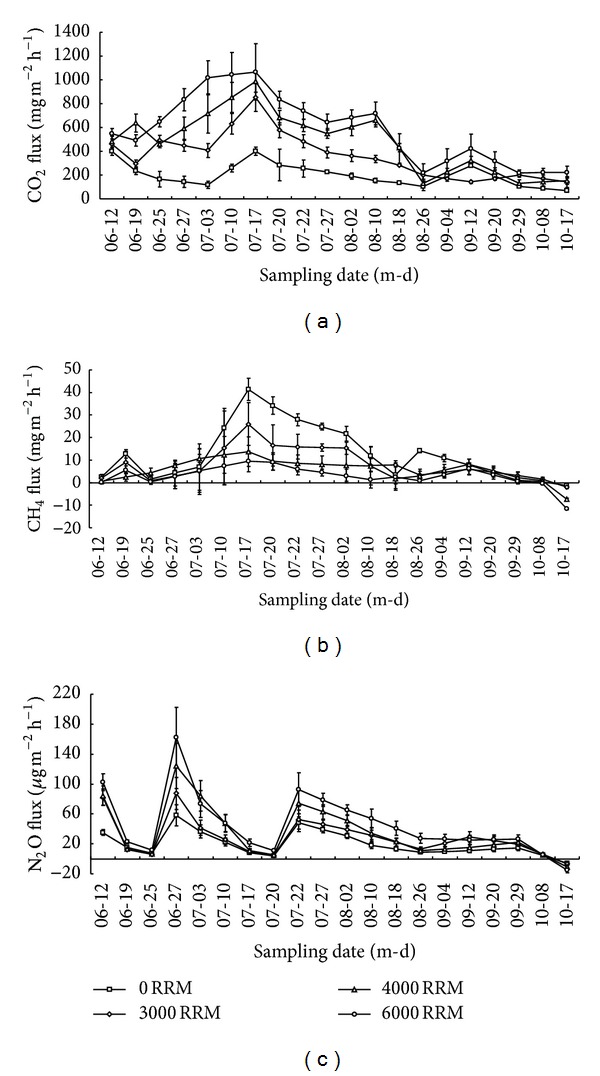
Changes in soil CO_2_ (a), CH_4_ (b), and N_2_O (c) fluxes from NT paddy fields under different residue mulching treatments during the rice-growing season. The vertical bars are standard deviations of the mean, *n* = 3.

The seasonal changes in soil CO_2_, CH_4_, and N_2_O fluxes from NT paddy fields under different residue mulching treatments during the 2010 rice-growing season are shown in Figure 2. During the rice-growing season, the fluxes ranged from 70.9 mg m^−2^ d^−1^ to 401.1 mg m^−2^ h^−1^ for CO_2_, from −7.38 mg m^−2^ h^−1^ to 41.4 mg m^−2^ h^−1^ for CH_4_, and from −5.76 *μ*g m^−2^ h^−1^ to 58.2 *μ*g m^−2^ h^−1^ for N_2_O from no residue mulching treatment. Moreover, the fluxes varied from 129.6 mg m^−2^ d^−1^ to 1066.6 mg m^−2^ h^−1^ for CO_2_, from −11.6 mg m^−2^ h^−1^ to 9.42 mg m^−2^ h^−1^ for CH_4_, and from −15.2 *μ*g m^−2^ h^−1^ to 162.5 *μ*g m^−2^ h^−1^ for N_2_O from residue mulching treatments. In addition, peaks in soil CO_2_ and CH_4_ fluxes were found during the tillering stage and several peaks in N_2_O fluxes were observed immediately after N fertilization. The peaks in soil CO_2_ fluxes could be attributed to the increased availability of substrates from root exudation or microbial decomposition of leftover plant residues during this stage [21]. Moreover, high total CH_4_ and CO_2_ fluxes during this period could be related to high air temperature (Figure 1). The mean weekly air temperature ranged from 19.7°C to 32.5°C during the rice-growing season, and the air temperature ranged from 20°C to 33°C from mid-June to September (Figure 1). High air temperature is beneficial to CH_4_ and CO_2_ production. Increase in N_2_O fluxes could be attributed to increased substrate availability from N fertilization [31].

In the present study, residue mulching significantly affected seasonal total CO_2_ emissions, in which the treatments of 3000 RRM, 4000 RRM, and 6000 RRM showed more total seasonal CO_2_ emissions than untreated control, by 73%, 136%, and 186%, respectively (Table 2). Soil CO_2_ fluxes are caused by complex interactions between climate and several biological, chemical, and physical properties of the soil [32]. Applying crop residues to cropland affects soil organic C pool, soil nutrients, and microbial environments and activities, thus influencing CO_2_ emissions [33]. Significant positive effects of residue mulching on soil CO_2_ emissions from NT paddy fields (Figure 2 and Table 3) indicate increased microbial activities resulting from high amounts of easily dissolved organic C from the decomposition of oilseed rape residues [34]. This expectation was confirmed in a previous, related study [35]. This previous study showed that CO_2_ emissions from soil amended with rice straw were significantly related to dissolved organic C (*r* = 0.95) and microbial biomass C (*r* = 0.94). In a field study conducted on a paddy sandy clay loam soil in eastern India, Bhattacharyya et al. [15] showed that soil treated with rice straw and green manure could produce more CO_2_ emissions than untreated soil. Bhattacharyya et al. [15] also found total organic C and microbial biomass C to be closely correlated with soil CO_2_ emissions. Similar results have been observed by other researchers [14, 20, 34]. The average depth of the water layer in the present study was approximately 4-5 cm during the rice-growing season. In this way, the top of the mulched oilseed rape residue was exposed to the atmosphere, leading to the oxidation of a substantial amount of CO_2_ produced by oilseed rape residue.

In this study, residue mulching had significant inhibiting effects on seasonal total CH_4_ emissions and the treatments of 3000 RRM, 4000 RRM, and 6000 RRM decreased total seasonal total CH_4_ emissions by 34%, 52%, and 75% compared with untreated control, respectively (Table 2). Previous studies have indicated that crop residue treatment does not only provide readily bioavailable organic C for CH_4_ production but also stimulates soil reduction and creates a strict reductive condition for CH_4_ production [36]. However, the present study showed residue mulching to inhibit CH_4_ emissions (Table 2). This finding is different from those of previous reports, which state that crop residues considerably increase CH_4_ emissions [8, 15, 37]. CH_4_ flux is a net product of three simultaneous processes, the production, oxidation, and transport of CH_4_. The position of crop residues directly influences CH_4_ production and oxidation and eventually affects CH_4_ emission from rice fields. Plots treated with residue mulching have greater dissolved organic C concentrations than untreated areas, possibly providing substrates for methanotrophic bacteria. However, residue mulch was exposed to more light, which suppressed methanogenesis. The subsequent growth of indigenous phototrophs was associated with the residue around the soil-floodwater interface [38]. The thin water layer (average 4-5 cm) observed during the rice-growing season caused the substantial CH_4_ produced from soil and crop residues to be oxidized before escaping to the atmosphere. Furthermore, crop residues on the NT soil surface blocked CH_4_ from the soil from entering the atmosphere. In this way, an elevation of O_2_ partial pressure in the soil-floodwater interface, caused by the method of irrigation used in the present study, led to CH_4_ oxidization. In this way, lower CH_4_ emissions were found to be attributable to residue mulching treatments in the present study. Another study conducted in the same part of China showed that rice straw mulching on NT paddy soil did not increase CH_4_ emissions from double rice cropping systems [16]. The present study indicated decreases in CH_4_ emissions associated with increasing crop residue rates (Figure 2 and Table 2). This finding is inconsistent with that of Naser et al. [39] who found positive linear relationships between CH_4_ emissions and the amount of straw used.

Nitrification and denitrification are two major microbial processes that are responsible for N_2_O emissions from paddy soil. Although nitrification is aerobic and denitrification is anaerobic, both processes have been known to occur simultaneously in paddy soil. Crop residues can provide readily available C, N, and other nutrients. In addition, this measure can increase organic C input of soil [34–36]. This can influence nitrification and denitrification rates and N_2_O emissions from the soil [34]. In the present study, the treatments of 3000 RRM, 4000 RRM, and 6000 RRM significantly increased total seasonal N_2_O emissions by 45%, 74%, and 128% over untreated soil, respectively (Table 2). Similar results were reported by Shan and Yan [7], who indicated that N_2_O emissions were higher when crop residues were mulched in paddy fields. Exposure of the mulching residue surface to the atmosphere led to high O_2_ concentration in the mulch. High O_2_ concentrations were found to stimulate nitrification and inhibit N_2_O reduction to N_2_ during denitrification [16, 29]. This increases N_2_O production. Second, the area of the soil/air interface in the present study can be enlarged by partial or complete spreading of mulches onto the field surface, thus favoring N_2_O production.

Soil N_2_O emissions are affected by the use of crop residues. These emissions are complex and dependent on residue quality, the time of residue application, the use of fertilizer, and soil and environmental conditions [29, 37]. Among these factors, the C/N ratio of the crop residues appears to be the primary regulator [7]. In general, crop residues with low C/N ratios have been found to decompose faster than residues with high C/N ratios [7]. Heal et al. (1997) [40] indicated that plant residues with C/N ratios <20 decompose rapidly and NH_4_^+^ is released through mineralization. Plant residues with intermediate C/N ratios of 25 to 75 can also decompose rapidly, but N mineralization activity is typically decreased by increased microbial immobilization. Residues with high C/N ratios (>75) are typically more difficult to break down than residues with low C/N ratios, and they generally stimulate net immobilization of soil available N, thereby decreasing the amount of N substrate available for N_2_O production [40]. In the present study, crop residues with high C/N ratios (48.5) were associated with temporary microbial immobilization of soil available N and with a decrease in N_2_O emissions resulting from reduced nitrification and denitrification. However, this immobilization of soil N could be counteracted by adding N fertilizers (210 kg N ha^−1^). In this way, higher N_2_O emissions were observed from plots treated with residue mulching than from untreated plots.

### 3.4. GWP, Net GWP, and GHGI

Residue mulching significantly affected GWPs, net GWP, and GHGI but did not affect rice grain yields (Table 3). GWP increased as the amount of mulching increased, but net GWP and GHGI decreased as the amount of mulching increased. The treatments of 3000 RRM, 4000 RRM, and 6000 RRM showed significantly more GWP than the control, by 9%, 23%, and 30%, respectively, but they showed less net GWP, by 33%, 50%, and 71%, respectively, and less GHGI, by 35%, 56%, and 72%, respectively.

When CH_4_ and N_2_O emissions from paddy fields are expressed as CO_2_ equivalents, the major contributor to GWP for the residue mulching treatments during the rice-growing season was clearly CO_2_, and not CH_4_, which only represented 12–36% of total GWP (Tables 2 and 3), thus indirectly reflecting the inhibitory effect of the residue mulching on CH_4_ emissions. In the present study, although residue mulching inhibited CH_4_ emissions from NT paddy fields, the stimulating effects of residue mulching on CO_2_ and N_2_O emissions, which overcame the reducing effects of residue mulching on CH_4_ emissions, had positive effects on GWPs (Tables 2 and 3).

Although residue mulching increased GWP from NT paddy fields, soil organic C sequestration from residue mulching might partially offset this increase. In this way, determining the degree to which residue mulching mitigates climatic impact requires an integrated perspective of the effects of residue on soil organic C sequestration. In the present study, residue mulching was found to have a mitigating effect on net GWP and GHGI (Table 3). This suggested that the practice of crop residue mulching with NT may be a good way to mitigate GHG emissions in central China without sacrificing rice grain yield. The present results differ from those found by Yao et al. [37]. They found that the use of wheat residue with NT increased net GWP and GHGI from rice-wheat rotation farmland. This discrepancy could be because of the different methods of residue application used and different durations of NT. In the study by Yao et al. [37], residues were incorporated, and NT was only applied during the wheat-growing season. Liu et al. [41] also reported that incorporating oilseed rape straws enhanced net GWP and GHGI during a rice-growing season in oilseed rape-rice rotation farming.

Although the present results indicated that net GWP and GHGI increased as the amount of residue mulch used increased, Qu et al. [28] reviewed the effects of the use of crop residue on rice grain yields in China and found that rice grain yield could decrease when the amount of residue used exceeded 11,250 kg ha^−1^. This is because increased concentrations of reducing matter from decomposition of large amounts of crop residue can inhibit rice growth. Accordingly, applying a rational amount of crop residue may mitigate GWP and maintain crop yield.

Although a field experiment conducted in Jurong of Jiangsu province, China, found that in-situ burning wheat straw decreased CH_4_ emissions from paddy soils due to decreased organic C provided by straw ash as substrate for CH_4_ production [36], the burning process also emitted a substantial amount of CH_4_ into the atmosphere [42], thus bringing about various adverse effects on the environment. NT is a simple cultivation technology that has attracted considerable attention since the establishment of a government policy favors the adoption of NT farming. In China, the research and the application of NT have developed quickly since the 1970s; by the end of 2008, NT had been applied to approximately 1.33 million hectares of land [1]. Therefore, it is urgent to manage increased crop residue for reducing environmental pollution caused by in-situ burning residue. In this study, although residue mulching on NT paddy fields increased CO_2_ and N_2_O emissions and GWP, this measure decreased net GWP and GHGI without decreasing rice grain yield. Therefore, it is advisable to advocate mulching of crop residue as a way to achieve agricultural economic viability and GHG mitigation from NT paddy fields.

GHG emissions are highly variable in time and space because of soil heterogeneity and climate variability [43]. For this reason, the outcome of the present study, which addressed a complete GHG accounting of GWP and GHGI as affected by residue mulching only during a rice-growing season after 3 years of the conversion of conventional tillage to NT under an oilseed rape-rice cropping system, is somewhat uncertain. Further study should be considered to determine residue mulching effects on GHG emissions from integrated oilseed rape—fallow—rice seasons after the long-term conversion of conventional tillage to NT. C emitted from the manufacturing and use of agricultural input, such as the use of pesticides, irrigation, and farm machinery, may negate all or part of the increased C sequestered by soil [23]. In this way, C emissions associated with changes in practices should be incorporated comprehensively into analyses of C sequestration [23].

## 4. Conclusions

The present study provided an insight into a complete GHG accounting of GWP, net GWP, and GHGI from NT paddy fields as affected by residue mulching during a rice-growing season after 3 years of oilseed rape-rice cultivation. Residue mulching on NT paddy fields was found to significantly increase CO_2_ and N_2_O emissions but decrease CH_4_ emissions. Residue mulching significantly increased GWP but decreased net GWP and GHGI due to increased soil organic C sequestration. Moreover, residue mulching did not decrease rice grain yields. Therefore, we conclude that residue mulching both limits GHG emissions and maintains rice grain yields if used with NT.

## Figures and Tables

**Table 1:** Differences in soil total organic C (g kg^−1^), organic C density (kg C ha^−1^), and sequestered organic C (kg C ha^−1^ season^−1^) during a rice-growing season from NT paddy fields under different residue mulching treatments.

Treatments	Total organic C concentrations before direct seeding	Total organic C concentrations at harvesting	Organic C density before direct seeding	Organic C density at harvesting	Sequestered organic C
0 RRM	18.4 ± 0.5^c^	18.5 ± 0.5^d^	46166^c^	46645^c^	479^d^
3000 RRM	19.5 ± 0.6^b^	20.0 ± 0.5^c^	49259^b^	50425^b^	1166^c^
4000 RRM	20.2 ± 0.7^a^	20.9 ± 1.76^b^	51008^a^	52744^ab^	1736^b^
6000 RRM	20.4 ± 1.1^a^	21.5 ± 0.9^a^	51476^a^	54104^a^	2629^a^

Different letters in a column mean significant differences at the 5% level. Values are the means ± SD, *n* = 3.

**Table 2:** Seasonal total emissions of CO_2 _(g m^−2^), CH_4_ (g m^−2^), and N_2_O (mg m^−2^) from NT paddy fields under different residue mulching treatments.

Treatments	CO_2_	CH_4_	N_2_O
0 RRM	570 ± 109^d^	34.8 ± 2.6^a^	54.8 ± 9.7^d^
3000 RRM	986 ± 126^c^	22.8 ± 3.3^a^	79.2 ± 8.5^c^
4000 RRM	1346 ± 239^b^	16.6 ± 3.6^c^	95.1 ± 10.0^b^
6000 RRM	1632 ± 313^a^	8.84 ± 3.1^d^	125.1 ± 9.4^a^

Different letters in a column mean significant differences at the 5% level. Values are the means ± SD, *n* = 3.

**Table 3:** GWP, net GWP, rice grain yield, and GHGI of different residue mulching treatments.

Treatments	GWP/ (kg CO_2_ equivalents ha^−1^)	Net GWP/ (kg CO_2_ equivalents ha^−1^)	Rice grain yield/ (kg ha^−1^)	GHGI/ (kg CO_2_ equivalents kg^−1^ grain yield)
0 RRM	14560 ± 1259^c^	8863 ± 1789^a^	7764 ± 190^a^	1.14^a^
3000 RRM	15800 ± 1920^b^	5936 ± 1264^ab^	8062 ± 179^a^	0.74^b^
4000 RRM	17898 ± 1648^ab^	4433 ± 477^c^	8835 ± 224^a^	0.50^c^
6000 RRM	18904 ± 2789^a^	2583 ± 436^d^	8134 ± 150^a^	0.32^d^

Different letters in a column mean significant differences at the 5% level. Values are the means ± SD, *n* = 3.
